# 

**Published:** 1902-01

**Authors:** 


### BOOKS RECEIVED.

Transactions of the American Otological Society at its Thirty-fourth Annual Meeting, held at the Pequot House, New London, Conn., July 16, 1901. Vol. XII., Part IV. Edited by S. B. St. John, Secretary protem. Published by the Society. New Bedford, Mass.: Mercury Publishing Co, Printers. 1901.

The Four Epochs of Woman's Life. A Study in Hygiene. By Anna M. Galbraith, M. D., Author of "Hygiene and Physical Culture for Women;" Fellow of the New York Academy of Medicine, etc With an Introductory Note by John H. Musser, M. D., Professor of Clinical Medicine, University of Pennsylvania. Duodecimo volume of 200 pages. Philadelphia and London: W. B. Saunders & Co. 1901. [Price, cloth, $1.25 net.]

Saunders's Question Compends. Essentials of Physiology. Prepared especially for Students of Medicine, and arranged with questions following each chapter. By Sidney P. Budgett, M. D., Professor of Physiology, Medical Department of Washington University, St. Louis. 16mo volume of 233 pages, finely illustrated with many full-page half-tones. Philadelphia and London: W. B. Saunders & Co. 1901. [Price, cloth, $1.00 net.]

The Practical Medical Series of Year Books. Ten volumes. Issued monthly, under the general editorial charge of Gustavus P. Head, M. D., Professor of Laryngology and Rhinology in the Chicago Post-Graduate Medical School. Vol. I. General Medicine. Edited by Frank Bellvyg, M. S., M D, Head of the Medical Department and Dean of the Faculty of Rush Medical College, Chicago. With the collaboration of S. C. Slanlen, M. D. October, 1901. Pp. 275. Chicago: The Year Book Publishers, 40 Dearborn Street. [Cloth, $1.50.]

Transactions of the American Laryngological Association at the Twenty-third Annual Meeting, held at New Haven, Conn., May 27, 28 and 29, 1901. Edited by the Secretary, James E. Newcomb, M. D. New York: The Roony & Otten Printing Co. 1901.

Transactions of the Medical Society of the State of New Jersey for 1901. Edited by William J. Chandler, M. D., Secretary. Newark: L. Q. Hardham, Printer.

An Experimental Research into Certain Problems Relating to Surgical Operations. An essay awarded the Alvarenga Prize for 1901, by the College of Physicians of Philadelphia. By George W. Cole, A. M., M. D., Ph. D., Professor of Clinical Surgery, Medical Department Western Reserve University; Assistant Surgeon to Lakeside Hospital, Cleveland. Philadelphia: J. B. Lippincott Co. 1901.

A Manual of Clinical Laboratory Methods. By John Benjamin Nichols, M. D., in Charge of the Clinical Laboratory of Garfield Hospital; Hematologist to Columbian Hospital; Professor of Normal Histology in the Medical Department of Columbian University, Washington, D. C. Illustrated. New York: William Wood & Co. 1901.

Gynecological Pathology. A Manual of Microscopic Technique and Diagnosis in Gynecological Practice. For Students and Physicians. By Dr Carl Abel, Privat-Docent, Berlin. Translated and edited by Samuel Wyllis Baudler, M. D., Adjunct Gynecologist to the Beth Israel Hospital, New York. With a chapter on the embryology of the female genitalia and the pathological growths developing from embryonal structures. Illustrated by 100 engravings. New York: William Wood & Co. 1901.

Progressive Medicine. Vol. IV., December, 1901. A Quarterly Digest of Advances, Discoveries and Improvements in the Medical and Surgical Sciences. Edited by Hobart Amory Hare, M. D., Professor of Therapeutics and Materia Medica in Jefferson Medical College of Philadelphia. Assisted by H. R. M. Landis, M. D., Assistant Physician to the Outpatient Medical Department of the Jefferson Medical College Hospital. Octavo, handsomely bound in cloth, 409 pages. Illustrated. Issued Quarterly. Philadelphia and New York: Lea Brothers & Co. 1901. [Price, $10 00 a year.]

Gaylord and Aschoff's Pathological Histology. The Principles of Pathological Histology. By Harvey R. Gaylord, M D., Professor of Surgical Pathology in the University of Buffalo, N. Y.; and Ludwig Aschoff, M. D., Professor and First Assistant in the Pathological Institute of the University of Göttingen, Germany. With an introductory note by William H. Welch, M. D., Professor of Pathology in Johns Hopkins University, Baltimore. In one very handsome quarto volume of 354 pages, with 81 engravings in the text and 40 full-page plates. Philadelphia and New York: Lea Bros & Co., Publishers. [Price, cloth, $7.50 net.]

Transactions of the American Surgical Association. Volume the Nineteenth. For the Year 1901. Edited by Richard H. Herte, M. D., Recorder of the Association. Pp. xxviii.—514. Philadelphia: William J. Dornan, Printer. 1901.

Transactions of the State Medical Society of Wisconsin. Fourth Year, 1901. Including Constitution and By-Laws. Edited by Charles S. Shelden, M. D., Secretary. Madison: State Journal Printing Co. 1901.

Lea's Series of Pocket Text-Books. Hayden on Venereal Diseases. A Pocket Text-Book of Venereal Diseases. For Students and Practitioners. By James R Hayden, M. D., Chief of Clinic and Instructor in Venereal and Genito-Urinary Diseases in the College of Physicians and Surgeons, New York, etc. New (3d) edition, thoroughly revised. In one handsome 12mo volume of 304 pages, with 66 engravings. Philadelphia and New York: Lea Brothers & Co., Publishers. [Cloth, $1.75 net; flexible leather, $2.25 net.]

Peru History of Coca. "The Divine Plant" of the Incas. With an introductory account of the Incas, and of the Andean Indians of today. By W. Golden Mortimer, M. D., Fellow of the New York Academy of Medicine; Member of the Medical Society of the County of New York; Member of the New York Academy of Sciences; Member of the American Museum of Natural History; formerly Assistant Surgeon to the New York Throat and Nose Hospital, etc. Octavo, pp. xxxii.—576. With 178 illustrations. New York: J. H. Vail & Co. 1901. [Price, $5.00 net.]

First Aid to the Injured and Sick. By F. J. Warwick, B. A., M. B., Cantab., Associate of King's College, London; Surgeon-Captain, Volunteer Medical Staff Corps, London Companies, etc.; and A. C. Tunstall, M. D., F. R. C. S. Ed., Surgeon-Captain Commanding the East London Volunteer Brigade Bearer Company; Surgeon to the French Hospital and to the Children's Home Hospital, etc. 16mo volume of 232 pages and nearly 200 illustrations. Philadelphia and London: W. B. Saunders & Co. 1901. [Price, cloth, $1.00 net.]

